# Severe Aplastic Anemia Complicated with Fatal Invasive Fungal Infections in a Young Patient Harboring Perforin Gene Polymorphisms

**DOI:** 10.3390/hematolrep17030025

**Published:** 2025-05-06

**Authors:** Maria I. Krithinaki, Ioannis Kokkinakis, Styliani Markatzinou, Christos Masaoutis, Elena Solomou, Ioanna Papakitsou, Nektaria Xirouchaki, Ioannis Liapis, Helen A. Papadaki, Charalampos G. Pontikoglou

**Affiliations:** 1 Department of Hematology, School of Medicine, University of Crete, University General Hospital of Heraklion, 71500 Heraklion, Greece; medp2012214@med.uoc.gr (M.I.K.); e.papadaki@uoc.gr (H.A.P.); 2 Department of Pathology, School of Medicine, University of Crete, 71500 Heraklion, Greece; kokkinakhsgianniskims@gmail.com; 3 Department of Medical Imaging, University General Hospital of Heraklion, 71500 Heraklion, Greece; markastella@yahoo.gr; 4 Department of Hemopathology, Evangelismos General Hospital, 11521 Athens, Greece; cmasaout@med.uoa.gr; 5 Internal Medicine-Hematology, University of Patras Medical School, 26500 Rion, Greece; esolomou@upatras.gr; 6 Department of Intensive Care Medicine, University General Hospital of Heraklion, 71500 Heraklion, Greece; medp2011875@med.uoc.gr (I.P.); nxirouchaki@pagni.gr (N.X.); 7 Department of Hematology, Aghios Georgios Hospital, 73300 Chania, Greece; aimatologiki@chaniahospital.gr

**Keywords:** severe aplastic anemia, perforin gene mutation, immunosuppressive therapy, aspergillosis, mucormycosis

## Abstract

Background: Severe aplastic anemia (SAA) is an uncommon life-threatening disorder characterized by hypocellular bone marrow and pancytopenia. It is typically associated with immune-mediated mechanisms, requiring immunosuppressive therapy (IST) or hematopoietic stem cell transplantation (HSCT). Infections, especially invasive fungal infections such as mucormycosis and aspergillosis, constitute principal causes of morbidity and mortality in patients with SAA. Genetic predispositions, including perforin (PRF1) polymorphisms, may further complicate disease outcomes by impairing immune function. Case report: We describe a case of a 36-year-old female patient diagnosed with SAA, for whom IST was considered, due to the unavailability of a matched sibling donor for HSCT. The patient presented with a feverish condition and deep neck space abscesses were revealed by imaging, caused by invasive aspergillosis. To prioritize infection control, IST was postponed and antifungal therapy with abscess drainage was initiated. However, aspergillosis progressed, despite aggressive and prompt treatment, and ultimately resulted in sepsis, multiorgan failure, and death. In addition, mucormycosis was confirmed post-mortem. Two heterozygous *PRF1* polymorphisms (c.272C>T and c.900C>T), were identified by genetic testing, which may have contributed to immune dysregulation and fungal dissemination. Conclusions: The complex interplay between managing SAA and addressing invasive fungal infections, which remain a leading cause of mortality in immunocompromised patients, is highlighted in this case. The latter emphasizes the importance of prompt diagnosis and targeted treatment to alleviate infection-related complications while maintaining care continuity for the hematologic disorder. The detection of *PRF1* polymorphisms raises questions about their implication in immune regulation and disease trajectory, emphasizing the need for further research in this field.

## 1. Introduction

Aplastic anemia (AA) is a rare disorder characterized by pancytopenia and a hypocellular bone marrow (BM) in the absence of significant dysplasia, abnormal infiltrate or marrow fibrosis [1,2]. In approximately 70% of cases, AA is acquired, with the majority being idiopathic [1,2,3,4], while the remaining cases are attributed to inherited BM failure syndrome [1,2,4]. Diagnostic features of AA include the presence of at least two of the following: hemoglobin (Hgb) < 10 g/dL, platelet (PLT) count < 50 × 109/L, or neutrophil count < 1.5 × 109/L [2,5].

In terms of risk stratification, AA can be classified into three categories [2,5,6,7]: (1) Severe AA (SAA): characterized by marrow cellularity below 25% and at least two of the following: (a) neutrophil count below 0.5 × 10⁹/L, (b) PLT count below 20 × 10⁹/L, or (c) reticulocyte count below 60 × 10⁹/L (using an automated reticulocyte count). (2) Very severe AA (VSAA): defined similarly to SAA, but with a neutrophil count below 0.2 × 10⁹/L. (3) Non-severe AA (NSAA): refers to AA cases that do not meet the criteria for SAA or VSAA [2,5,6,7].

As the majority of acquired aplastic anemia cases, particularly those that are severe and acute, are believed to be immune-mediated, the conventional treatment for SAA/VSAA includes IST with antithymocyte globulin (ATG), cyclosporine (CSA) and eltrombopag [2,7,8]. Hematopoietic cell transplantation from a matched related donor is also considered a standard curative approach in eligible patients [2,7,8]. Emerging therapeutic approaches, although not routinely used in the upfront setting, include hematopoietic stem cell transplantation from matched unrelated donors and haploidentical donors [2,7,8]. In selected cases of NSAA, less intensive immunosuppressive treatment consisting of ATG and CSA, may be employed [2,7,8].

Infections are the leading cause of death in AA [9,10,11,12], where neutropenia—often prolonged—significantly increases the risk of invasive fungal infections and bacterial sepsis. Invasive fungal infections, particularly those caused by *Aspergillus* species, are a primary cause of mortality in AA patients [9,10,13,14]. Interestingly, *Zygomycetes* (*Mucor*, *Rhizopus* spp.), often clinically reported as “mucormycosis”, are the second leading cause of invasive fungal infections in AA [10,15,16,17].

We report a patient with SAA in whom genetic screening to exclude an inherited BM failure (IBFM) syndrome revealed polymorphisms in *PRF1*, a gene encoding perforin, previously associated with adverse prognosis [18]. The patient’s course was complicated by aspergillosis and mucormycosis, resulting in death on day 51 of admission.

## 2. Case Presentation

**Figure 1:**
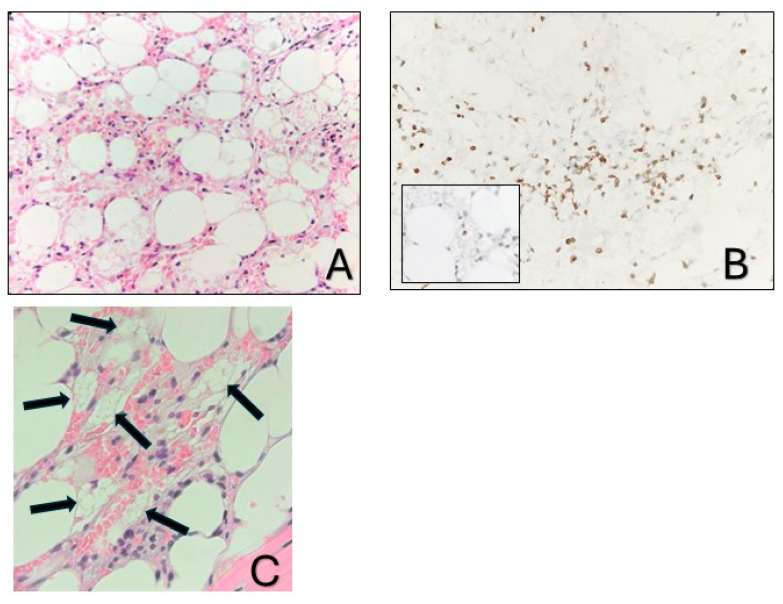
Bone marrow biopsy. (**A**). Markedly hypocellular bone marrow with stromal edema (hematoxylin-eosin stain; ×400). (**B**). The predominant cell population corresponds to T cells (CD3, ×400), with rare cytotoxic cells (inset: Granzyme B, ×400). (**C**). The arrows depict macrophages with foamy cytoplasm.

**Figure 2:**
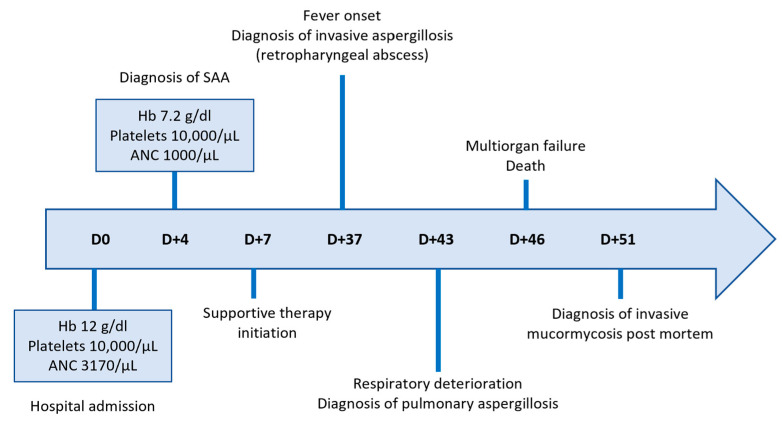
Timeline of key clinical events and hematologic parameters.

A 36-year-old Greek female patient, mother of two, with no significant medical or family history presented in the emergency department of a regional Greek hospital due to bruising on the trunk and the extremities. She was not taking any medication and had no history of recent infections. Nevertheless, she reported inhaling fumes from floor-cleaning solutions one day prior to her presentation. On examination, the patient had normal vital signs and was alert. Ecchymoses and petechiae were noted on the face, trunk and extremities, as well as soft palatal purpura. There was no peripheral lymphadenopathy, hepatomegaly or splenomegaly. Laboratory tests revealed a white blood cell count of 4100/μL (4000–10,000/μL), an absolute neutrophil count of 3170/μL (1750–8500/μL), and an absolute lymphocyte count of 800/μL (1500–4000/μL). Hgb was 12 g/dL (11.5–15.5 g/dL) with a reticulocyte count of 10 × 10^9^/L. PLT count was 10,000/μL (150,000/μL–450,000/μL). Examination of the peripheral blood smear confirmed the profound decrease in PLT numbers and showed a slight increase in PLT size, while both white and red blood cells appeared normal. There were no signs of hemolysis, and coagulation parameters, glucose levels, renal and liver function tests were all within normal limits. Serum immunoglobulin levels and thyroid function tests were also within the normal range. Serologic testing for Hepatitis B virus, Hepatitis C virus, Human Immunodeficiency Virus, Parvovirus B19, Herpes Simplex Virus, Varicella-Zoster Virus, Cytomegalovirus, and Epstein–Barr Virus was negative. Antinuclear antibody screening and the direct Coombs test were also negative. Based on these findings, a diagnosis of immune thrombocytopenia (ITP) was made, and the patient was treated with intravenous immunoglobulin and corticosteroids. On day +4, not only did PLT counts fail to increase, but the Hgb levels also declined (7.2 gr/dL) and the absolute neutrophil count (1000/μL) decreased. Bone marrow aspiration demonstrated marked hypocellularity, which was further confirmed by bone marrow trephine biopsy. The biopsy (Figure 1A–C) revealed a predominant population of T lymphocytes, which were scarce overall, with rare Granzyme-B-expressing cells, as well as a few polyclonal plasma cells, macrophages and mast cells. Rare granulocytic cells were also observed, while megakaryocytic and erythroid lineages were absent. CD34 immunohistochemistry was negative, and stromal edema was present. These findings raised the suspicion of SAA (Figure 2). *PRF1* sequencing revealed two heterozygous polymorphisms: c.272C>T (p.A91V; rs35947132) and c.900C>T (p.H300H; rs885822). According to ClinVar (RCV000547554.3; accessed 24 April 2025), the *PRF1* p.A91V variant exhibits inconsistent pathogenicity interpretations, with submissions classifying it from benign to unknown significance. The *PRF1* p.H300H variant is a synonymous alteration that is uniformly categorized as benign in ClinVar.

From day +7 until day +37, the patient continued inpatient care at the referring regional hospital, receiving supportive therapy consisting of PLT and red blood cell transfusions, epoetin zeta 40,000 IU/week, G-CSF 300 mcg o.d. and eltrombopag 150 mg o.d (Figure 2). According to international guidelines, as the patient was medically fit and under 40 years of age, she should have proceeded directly to hematopoietic stem cell transplantation if a histocompatibility leukocyte antigen (HLA) matched sibling donor had been identified. However, HLA typing failed to identify a suitable related donor and since the search for an unrelated donor was expected to take at least 8 weeks, the patient was transferred to the Hematology Department of a tertiary Greek hospital for immunosuppressive treatment with ATG and CSA.

**Figure 3:**
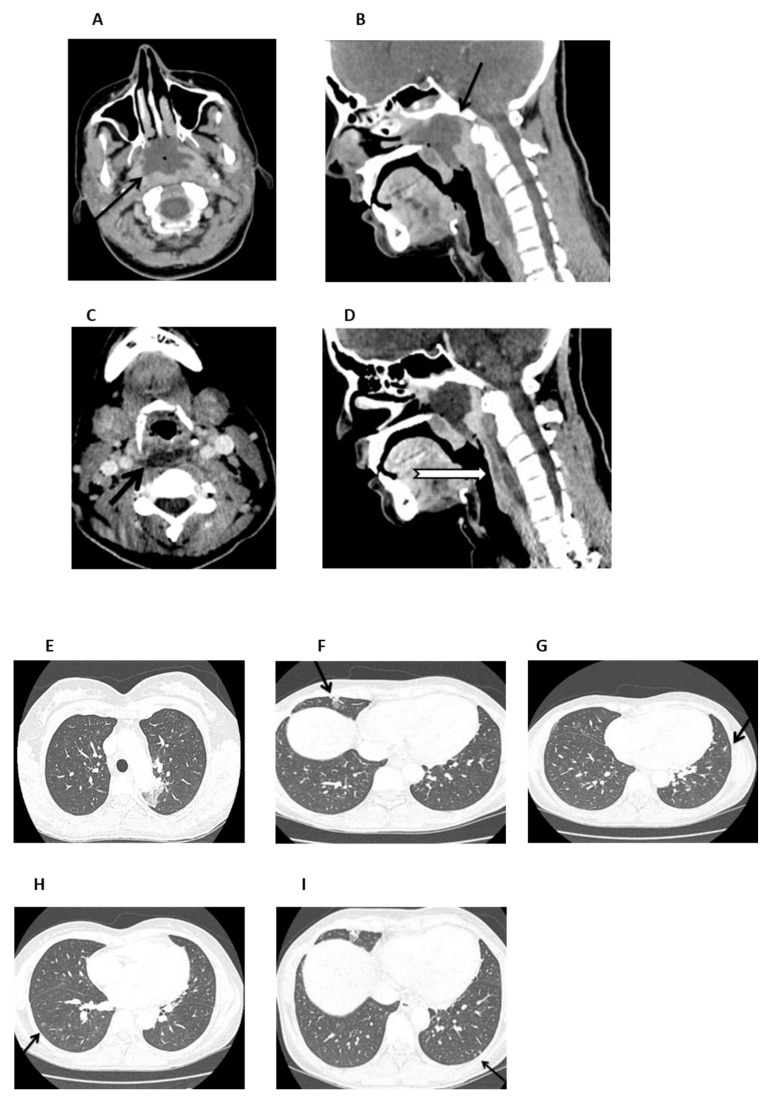
Neck and chest CT scan. (**A**,**B**). An axial and mid-sagittal image showing a large fluid collection of low attenuation with surrounding rim-enhancement (arrow) and some gaseous foci in the posterior aspect of the nasopharynx (arrow), measuring 3.3 × 4.1 × 2.8 cm, consistent with abscess formation. (**C**,**D**). An axial mid-sagittal image depicting another abscess in the retropharyngeal space (arrow) extending from the level of C2 vertebral body, to the C6 level with a craniocaudal diameter of 6.3 cm and a maximum depth of 1 cm, with a significant fat stranding extending into the visceral and carotid spaces, bilaterally. (**E**,**F**). Axial images depicting a small consolidation in the apicoposterior segment of the left upper lobe (arrow) and a lobar consolidation with ground-glass opacification in the right middle lobe (arrow). (**G**). Axial image showing centrilobular nodules with a linear branching pattern (tree-in-bud) in the lower lobes and in the lingula (arrow). (**H**,**I**) Axial images showing nodules up to 5 mm, some of them with a ground-glass halo, in the lower lobes (arrow), predominantly peripheral in distribution.

On admission (day +37), the patient was febrile and ill-appearing (Figure 2). She complained of a sore throat and difficulty in swallowing and was noted to have a “hot potato” voice. A deep-neck space infection was suspected and urgent ear–nose–throat (ENT) consultation and imaging studies were ordered. Head CT scan was normal, whereas neck CT scan revealed both nasopharyngeal and retropharyngeal abscesses, with the latter extending into the deep neck spaces (Figure 3A–D). A chest CT scan demonstrated consolidations in the left upper lobe and right middle lobe (Figure 3E,F), centrilobular nodules with tree-in-bud pattern (Figure 3G), along with few predominantly peripheral nodules, some of them with a ground-glass halo (Figure 3H,I).

**Figure 4:**
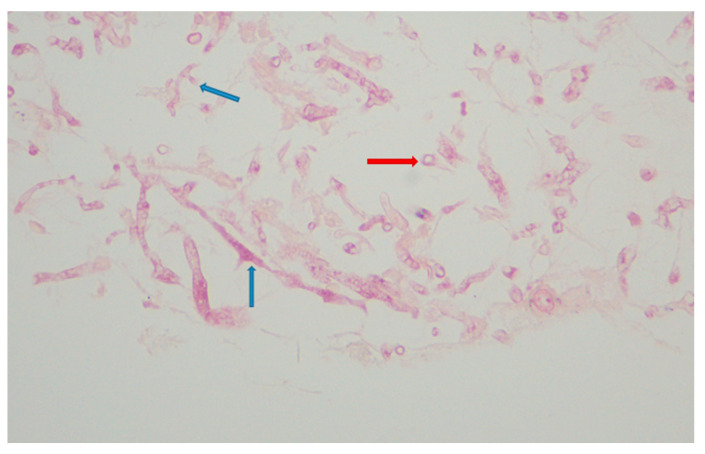
*Aspergillus* spp. Histopathology. Hyphae with acute branching angle (<45°) or dichotomous branching septate hyphae (blue arrows) and fragments of hyphae (red arrow) (hematoxylin-eosin stain; ×400).

Urgent abscess drainage, on day +37, revealed a loose formation with central necrosis, whereas pharyngeal puncture at the level of the epiglottis, to detect any residual abscess tissue, was negative. The patient was transferred to the Intensive Care Unit where she remained intubated under mechanical ventilation. She was febrile and hemodynamically unstable and received empiric broad-spectrum antibiotic therapy consisting of meropenem 2 gr t.i.d. and daptomycin 500 mg o.d., as well as corticosteroids for sepsis. The histopathologic results (rapid tissue biopsy) showed the presence of fungal hyphae (Figure 4) suggestive of *Aspergillus* spp. and the positive result of the Aspergillus-specific PCR assay in the nasophaygeal samples further supported this diagnosis (Figure 2). Antifungal therapy with isavuconazole 200 mg t.i.d. plus micafungin 100 mg o.d. was initiated on day +38.

As the fever persisted, and the general condition of the patient progressively deteriorated, antibiotic escalation was performed on day +39: meropenem was discontinued and ceftazidime tazobactam 2.5 gr t.i.d. and colistin 4.500.000 units b.i.d. were added. Of note, regular endoscopic examinations by the otolaryngologists failed to demonstrate any new signs of infection. On day +42, the patient developed lower quadrant tenderness, and bedside ultrasound revealed a large splenic infarct, which was confirmed by an abdominal CT scan. Because of this finding and as eltrombopag is rarely associated with thromboembolism [19], its administration was discontinued and the patient was maintained on PLT transfusions. A neck CT scan demonstrated extension of the fluid collection at the site of the previously treated surgical abscess into the nasal cavity and paranasal sinuses, while the retropharyngeal fluid showed a mild reduction in its craniocaudal diameter. Disease progression was also observed in a chest CT scan, including worsening consolidation in the left upper lobe, the appearance of a new smaller consolidation in the right upper lobe, and increased ground-glass opacities and solid nodules scattered throughout both lungs. These findings were not specific but could be due to a fungal etiology. Indeed, *Aspergillus*-specific PCR assay from the bronchoalveolar lavage on day +43 confirmed the diagnosis of invasive pulmonary aspergillosis (Figure 2).

Due to the high risk of hemorrhage, further surgical intervention was deemed inappropriate. The patient was instead maintained on antibiotic treatment and underwent regular transnasal drainage of the nasopharynx. Immunomodulatory treatment as salvage therapy for the underlying aplastic anemia was considered but ultimately rejected due to the presence of active infection. On day +45, a lung ultrasound was performed to investigate worsening respiratory failure, which confirmed progression of findings from the previous CT scan. Additionally, the patient was diagnosed with acute kidney injury and acute coronary syndrome. Cardiac ultrasound showed significantly reduced left ventricular contractility with signs of diffuse hypokinesia, most likely diagnostic of septic cardiomyopathy. Despite intensive management, the patient’s condition deteriorated rapidly over the next few hours, and she succumbed to sepsis and multiorgan dysfunction on day +46 (Figure 2). Notably, nasopharyngeal cultures returned for *Mucor* spp. five days after her death, suggesting aspergillosis and mucormycosis coinfection (Figure 2).

## 3. Discussion

We describe a case of a 36-year-old female patient who was diagnosed with SAA in a regional hospital of Crete. As the patient was medically fit and younger than 40 years old, allogeneic HSCT from a matched sibling donor should have been performed upfront, according to current practice [2,20,21] Yet, HLA typing did not identify a suitable related donor. In light of recent studies and evolving guidelines, upfront matched unrelated donor (MUD) HSCT was also considered as a viable option [2,7,22]. However, this option requires that a suitable donor be readily available [2,7,22], which was not the case. Indeed, the procedure of selecting an appropriate unrelated donor and completing the requisite preliminary stages for transplantation was expected to take a minimum of eight to twelve weeks. Due to the urgency of treatment initiation, the patient was transported to the Hematology Department of a tertiary hospital to begin immunosuppressive medication with ATG and CSA with the addition of eltrombopag, based on the existing literature [2,23]. On admission, though, the patient presented with fever and appeared clinically unwell. Deep neck space abscesses were identified and attributed to invasive aspergillosis. This prompted urgent drainage and the initiation of fungal and antibiotic therapy. Immunosuppressive treatment was deferred in order to control infection and achieve defervescence [2,20]

Infections are the leading cause of mortality in individuals with AA [9,10,11,12]. A retrospective study of patients with aplastic anemia (AA) admitted to the National Institutes of Health between 1978 and 1990 reported that 62% of patient deaths were attributed to infections, with invasive fungal infections caused by *Aspergillus* spp. and *Candida* spp. accounting for 50% of these infection-related death [9]. Similarly, a study conducted at the MD Anderson Cancer Center reported that between 1994 and 2000, 42% of AA-related deaths were attributed to fungal infections, predominantly mold infections [13]. In contrast, a more recent retrospective study from Thailand found that between 2010 and 2015, only 16.9% of patients with AA died from fungal infections [24]. This decrease in fungal-infection-related mortality is consistent with the observed reduction in the prevalence of these infections in patients with AA after the year 2000 [10]. Across all these studies, the neutropenia associated with AA, particularly when severe and prolonged, was documented to significantly increase the risk of infections [9,10,11,12,13].

As invasive aspergillosis and mucormycosis are life-threatening fungal infections in immunocompromised patients [25,26], early diagnosis is crucial for improving prognosis. However, this remains a significant challenge, requiring a high index of suspicion, thorough assessment of host risk factors, and prompt evaluation of clinical manifestations, which are often nonspecific [25,26]. In the case reported here, the patient’s neutropenia—a key predisposing factor for invasive fungal infections [27,28]—heightened our vigilance when the possibility of a deep-neck space infection was considered. As Galactomannan testing—a serum assay for detecting a polysaccharide cell wall component of *Aspergillus* spp. [29]—was not available at the time of hospitalization, the diagnosis of invasive aspergillosis was established through histopathological examination. Specimens from the necrotic formation removed from the nasopharynx revealed typical dichotomous and septate hyphae consistent with *Aspergillus* spp. [30]. The diagnosis was further supported by a positive *Aspergillus* PCR test from the nasopharyngeal samples, while PCR testing of BAL provided evidence for invasive pulmonary aspergillosis. Interestingly, cultures from both the nasopharyngeal abscess and BAL were negative for *Aspergillus* spp., reflecting the low sensitivity of this diagnostic method [29]. However, tissue cultures from the nasopharynx tested positive for *Mucor* spp., five days after the patient’s death, despite the absence of identifiable hyphae in the histopathological examination. Pulmonary imaging studies were non-specific. This highlights the importance of employing multiple diagnostic approaches when assessing a patient for invasive fungal infection.

The treatment of invasive aspergillosis in our patient consisted of drainage of the nasopharyngeal abscess and combination antifungal therapy. Unfortunately, the infection progressed at the nasopharynx and paranasal sinuses and in the lungs, as evidenced by the respective imaging studies 6 days later. Surgical debridement, as adjunctive therapy for Aspergillus rhinosinusitis [31,32], was considered but ultimately rejected due to concerns regarding surgical complications, particularly hemorrhage, given the patient’s severe thrombocytopenia.

AA is a diagnosis of exclusion that requires ruling out other causes of pancytopenia with hypocellular BM, including ΙBFM, especially in patients aged < 40 or with a positive family history and suggestive physical features [2,4]. Within this context, AA must be distinguished from hemophagocytic lymphohistiocytosis (HLH) [18,33].

HLH is a rare, rapidly progressive and potentially life-threatening syndrome characterized by extreme immune system activation that can present with pancytopenia and usually manifests as a febrile disorder associated with multiorgan dysfunction [34,35]. HLH is classified as either primary (familial), due to inherited genetic mutations, or secondary, triggered by an abnormal immune response to infection, malignancy, or autoimmune disease [35].

The etiological association of mutations of the PRF1 gene with familial HLH has been well established [34,35]. Perforin, a protein stored in the secretory granules in CD8+ T cells and natural killer (NK) cells, is critically implicated in their cytotoxic activity [36]. Upon activation, these lymphocytes release perforin, which subsequently forms pores in the membrane of the target cell, resulting in osmotic lysis. In addition, perforin facilitates the delivery of the proapoptotic serine protease granzyme B, also stored in cytotoxic lymphocyte granules, into the target cell triggering apoptosis [36,37].

Germline genetic testing in our patient revealed the presence of two heterozygous *PRF1* polymorphisms: c.272C>T (p.A91V; rs35947132) and c.900C>T (p.H300H; rs885822). The presence of PRF1 genetic alterations in AA patients has been previously documented in two studies [18,38]. In the study by Solomou et al., *PRF1* variants were identified in 5 out of 75 AA patients [18]. Three of these patients harbored the A91V and H300H polymorphisms and two of them exhibited hemophagocytosis in the bone marrow but did not display the other typical features of HLH. Interestingly, no hemophagocytosis could be detected in our patient despite the presence of macrophages in the BM. It has been postulated that *PRF1* variants may be associated with a more severe form of AA, as patients harboring these variants either failed to respond to immunosuppressive therapy or demonstrated only a partial response [18]. Another study by Rafati et al. [38] employed whole-exome sequencing to assess the frequency of gene variants in 14 HLH-associated genes in a cohort of 684 patients with SAA who had undergone unrelated donor HSCT. In that study, the *PRF1* variant c.272C>T (p.A91V; rs35947132) was detected in 8.5% (58 of 684) of SAA patients. Yet, in contrast to the findings of Solomou et al. [18], it was not associated with a worse post-HSCT survival.

Pre-clinical studies have shown that perforin is crucial for primary immunity against specific fungal infections. For instance, mice lacking perforin demonstrate heightened mortality and an elevated fungal load after infection with Histoplasma capsulatum [39]. Furthermore, previous reports have demonstrated that perforin plays a critical role in natural killer (NK)-cell-mediated antifungal activity against invasive mold infections [40]. In particular, in vitro evidence demonstrates that pretreatment of human NK cells with concanamycin A, which accelerates perforin degradation, significantly reduces hyphal damage caused by *Aspergillus fumigatus* and *Rhizopus oryzae*, whereas exposure to purified perforin enhances hyphal killing [41,42].

Patients with AA carrying PRF1 genetic alterations have been reported to exhibit low or absent perforin levels and significantly impaired NK cell cytotoxicity [18]. In addition, heterozygous healthy carriers of the PRF1 p.A91V variant (A91V/+) have been shown to exhibit at least a 35% reduction in NK cytotoxicity compared with wild-type individuals [43]. Our patient was heterozygous for the PRF1 c.272C>T (p.Ala91Val; rs35947132) variant, whose pathogenicity classification in ClinVar ranges from benign to uncertain significance. She also harbored the synonymous PRF1 c.900C>T (p.H300H) variant, which is deemed benign. We recognize that individuals with SAA are inherently at high risk for invasive fungal infections and may succumb to them regardless of underlying genetic predisposition [9,10,11,12]. However, based on previous reports linking the p.A91V variant with reduced NK cell cytotoxicity [40,43], we hypothesize that its presence may have impaired antifungal immune responses in this patient, potentially increasing her susceptibility to the spread of invasive aspergillosis and its lethal consequences. This interpretation remains controversial, especially as a large study could not establish a definitive disease association with this variant [44]. Further research is warranted to shed light on the clinical relevance of the p.A19V variant in the context of infection susceptibility

## 4. Conclusions

We present the case of a 36-year-old female patient diagnosed with SAA who was transferred to the Hematology Department of the University General Hospital of Heraklion (PAGNI) for IST due to the absence of a matched sibling donor for allogeneic HSCT. The patient presented with fever, and deep neck space abscesses were identified, attributed to invasive aspergillosis. IST was postponed to treat the infection, which was further complicated by mucormycosis. Aspergillosis infection progressed and involved the lungs and paranasal sinuses, ultimately leading to the patient’s death, despite the prompt initiation of surgical drainage of the abscess and antifungal therapy.

This case highlights the significant difficulties in the management of SAA, especially in the presence of life-threatening fungal infections, which necessitate balancing the need for IST with infection control. It also underscores the need for prompt and aggressive diagnostic and therapeutic modalities to manage infections in immunocompromised patients. Finally, the detection of *PRF1* polymorphisms in this patient raises important questions about their potential impact on immune dysregulation and disease progression, emphasizing the necessity for further research in this area.

## Data Availability

The authors confirm that the data supporting this study are available in this article.

## Abbreviations

The following abbreviations are used in this manuscript:

AA	Aplastic Anemia
ATG	Antithymocyte Globulin
BM	Bone Marrow
BMF	Bone Marrow Failure
CSA	Cyclosporine
ENT	Ear-Nose-Throat
GCSF	Granulocyte Colony-Stimulating Factor
Hgb	Hemoglobin
HLA	Histocompatibility Leukocyte Antigen
HLH	Hemophagocytic Lymphohistiocytosis
HSCT	Hematopoietic Stem Cell Transplantation
IBFM	Inherited Bone Marrow Failure
IST	Immunosuppressive Therapy
ITP	Immune Thrombocytopenia
NK	Natural Killer
NSAA	Non-Severe Aplastic Anemia
PCR	Polymerase Chain Reaction
PLT	Platelet
PRF1	Perforin
SAA	Severe Aplastic Anemia
VSAA	Very Severe Aplastic Anemia
